# PIM2-mediated phosphorylation of hexokinase 2 is critical for tumor growth and paclitaxel resistance in breast cancer

**DOI:** 10.1038/s41388-018-0386-x

**Published:** 2018-07-09

**Authors:** Tingting Yang, Chune Ren, Pengyun Qiao, Xue Han, Li Wang, Shijun Lv, Yonghong Sun, Zhijun Liu, Yu Du, Zhenhai Yu

**Affiliations:** 1 Department of Reproductive Medicine, Affiliated Hospital of Weifang Medical University, Weifang, Shandong Province P.R. China; 2 Department of Pathology, Affiliated Hospital of Weifang Medical University, Weifang, Shandong Province P.R. China; 3 Department of Medical Microbiology, Weifang Medical University, Weifang, Shandong Province P.R. China

## Abstract

Hexokinase-II (HK2) is a key enzyme involved in glycolysis, which is required for breast cancer progression. However, the underlying post-translational mechanisms of HK2 activity are poorly understood. Here, we showed that Proviral Insertion in Murine Lymphomas 2 (PIM2) directly bound to HK2 and phosphorylated HK2 on Thr473. Biochemical analyses demonstrated that phosphorylated HK2 Thr473 promoted its protein stability through the chaperone-mediated autophagy (CMA) pathway, and the levels of PIM2 and pThr473-HK2 proteins were positively correlated with each other in human breast cancer. Furthermore, phosphorylation of HK2 on Thr473 increased HK2 enzyme activity and glycolysis, and enhanced glucose starvation-induced autophagy. As a result, phosphorylated HK2 Thr473 promoted breast cancer cell growth in vitro and in vivo. Interestingly, PIM2 kinase inhibitor SMI-4a could abrogate the effects of phosphorylated HK2 Thr473 on paclitaxel resistance in vitro and in vivo. Taken together, our findings indicated that PIM2 was a novel regulator of HK2, and suggested a new strategy to treat breast cancer.

## Introduction

High rate of aerobic glycolysis is a hallmark of cancers which has promoted the development of aerobic glycolysis inhibitors and other novel drugs targeting metabolic enzymes to treat cancer [1]. Hexokinase (HK) is the first step of aerobic glycolysis that generates glucose-6-phosphate (G6P), which is further used to produce two ATP molecules. G6P enters into the pentose phosphate pathway to produce NADPH and anabolic intermediates [2]. There are four major hexokinase isoforms in mammalian tissues, including HK1, HK2, HK3, and HK4 (also known as glucokinase), and the high-affinity HK1, HK2, and HK3 isoforms are inhibited by excess G6P, except for the low-affinity HK4 isoform, which is mainly expressed in the pancreas and liver [3]. Compared to other isoforms, HK2 expression is upregulated in many types of tumors associated with enhanced glucose flux [4]. Furthermore, HK2 is required for tumor initiation and maintenance, and is a key mediator of aerobic glycolysis, promoting tumor metastasis and growth in many types of cancers [2, 5, 6**]**. Recent studies showed that HK2 played an important role in glycolysis, as well as other functions. HK2 binds to the autophagy suppressor, mTOR complex 1 (TORC1), and positively regulates glucose starvation-induced autophagy which is crucial for cell survival [7, 8]. HK2 is not only mediated by levels of mRNA and proteins, which are due to its post-translational modifications. The phosphorylation of HK2 at Thr473 by AKT promotes its mitochondrial association to protect cardiomyocytes [9], and AKT inhibitor can decrease the location of HK2 to the outer mitochondrial membrane to inhibit glycolysis in tumor cells [5]. However, the post-translational modifications of HK2 that contribute to the effects of glycolysis are still unknown.

The Proviral Insertion in Murine Lymphomas 2 (PIM2) kinase belongs to serine/threonine kinase family composed of another two isoforms, PIM1 and PIM3, which are highly conserved and constitutively activated [10]. PIM2 shares nearly 60% homology with PIM1 and PIM3, which play important roles in crucial signals pathways, including cell proliferation, migration, apoptosis, survival, and metabolism [10]. PIM2 is upregulated in many malignancies, and is transcriptionally mediated by the Janus kinase/signal transducers and activators of transcription (JAK/STAT) and nuclear factor-κB pathways [11]. PIM2 as a serine/threonine kinase that exerts its functions mainly through phosphorylation of its substrates [11]. PIM2 phosphorylates TSC2 on Ser1798 and relieves the suppression of TSC2 on mTOR-C1 which could be a promising therapeutic target for multiple myeloma [12]. Moreover, PIM2 directly phosphorylates FOXP3, leading to decreased suppressive functions of Treg cells [13]. Although the oncogene functions of PIM2 are important for cancer cells, the mechanisms of regulation of glycolysis remain uncharacterized.

In the present study, we used in vivo and in vitro biochemical methods to determine if PIM2 directly bound to HK2, and phosphorylated HK2 on Thr473. PIM2 enhanced HK2 protein stability through a chaperone-mediated autophagy (CMA) pathway. Moreover, the expression levels of PIM2 and pThr473-HK2 were positively correlated in breast cancer tissues. Importantly, phosphorylation of HK2 on Thr473 promoted glycolysis and was required for breast cancer cell growth in vitro and in vivo. Furthermore, phosphorylation of HK2 on Thr473 promoted autophagy in glucose starvation, and enhanced paclitaxel resistance in vitro and in vivo. These data provided the rationale for further use of the PIM2–HK2 pathway as a potential target for therapeutic intervention in breast cancer.

## Results

### PIM2 interacts with HK2

**Fig. 1:**
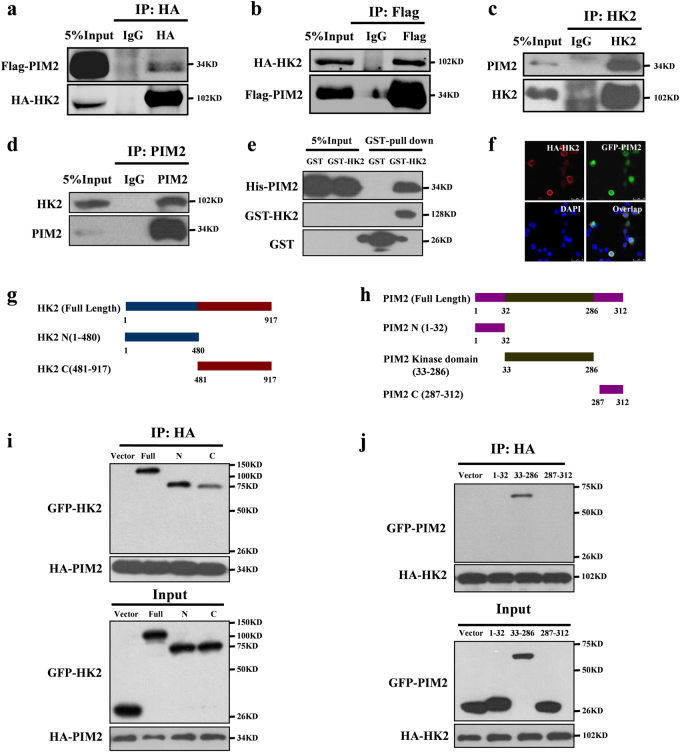
PIM2 interacts with HK2. **a–e** Immunoprecipitation and immunoblotting analyses were performed with the indicated antibodies. **a**, **b** HEK293T cells were overexpressed the indicated HA-tagged HK2 and Flag-tagged PIM2 proteins. Immunoprecipitations with an anti-HA antibody (**a**) or anti-Flag antibody (**b**) were performed. **c**, **d** Immunoprecipitations with an anti-PIM2 antibody (**c**) or anti-HK2 antibody (**d**) were performed using cell lysate from MCF-7 cells. **e** Purified GST-tagged HK2 or GST was mixed with His-PIM2 for GST-pulldown assay. **f** Confocal immunofluorescence microscopy was performed to analyze localization of HK2 and PIM2 in MCF-7 cells. **g** The HK2 truncation mutants used in this study. **h** HEK293T cells were overexpressed the indicated HA-tagged PIM2 and GFP-tagged HK2 fragments proteins. Immunoprecipitation with an anti-HA antibody was performed. **i** The PIM2 truncation mutants used in this study. **j** HEK293T cells were overexpressed the indicated HA-tagged HK2 and GFP-tagged PIM2 fragments proteins. Immunoprecipitation with an anti-HA antibody was performed

PIM2 plays an important role in regulating cell glycolysis, proliferation, and survival [11, 14**–**16]. To determine the mechanisms underlying PIM2 functions in breast cancer, we performed mass spectrometry analyses of the immunoprecipitated PIM2 complex in MCF-7 cells, and found that HK2 was associated with PIM2 (Supplementary Fig. 1a and Supplementary Table 1). This association was confirmed by co-immunoprecipitation (Co-IP) using overexpressed HA-tagged HK2 and Flag-tagged PIM2 in HEK293T cells (Fig. 1a, b). Moreover, the endogenous PIM2 also interacted with endogenous HK2 in MCF-7 cells (Fig. 1c, d). As shown in Fig. 1e, GST-tagged HK2 could pulldown His-tagged PIM2, which suggested that PIM2 could directly interact with HK2. Furthermore, immunofluorescence confocal microscopy analyses showed that PIM2 mainly overlapped with HK2 in the cytoplasm of MCF-7 cells (Fig. 1f). Interestingly, we found overexpressed HK2 interacted with PIM2, but failed to interact with PIM1 and PIM3 (Supplementary Fig. 1b). Moreover, we also demonstrated that PIM2 kinase activity was dispensable for their interaction (Supplementary Fig. 2a). Together, our results demonstrated that PIM2 physically interacted with HK2.

To identify the domain(s) of HK2 responsible for interacting with PIM2, we generated two HK2 fragments: GFP-tagged N-HK2 (amino acid residues 1–480) and GFP-tagged C-HK2 (amino acid residues 481–917) (Fig. 1g), and performed Co-IP assays. As shown in Fig. 1i, PIM2 strongly bound to the N-terminal domain of HK2. Using a similar approach, we generated three additional GFP-tagged proteins containing amino acid residues 1–32, 33–286, or 287–312 of PIM2 (Fig. 1h), and found that HK2 specifically interacted with PIM (33–286), belonging to the kinase domain (Fig. 1j). Taken together, the results indicated that the N-terminal domain of HK2 and the kinase domain of PIM2 were required for their interaction.

### PIM2 phosphorylates HK2 at Thr473

**Fig. 2:**
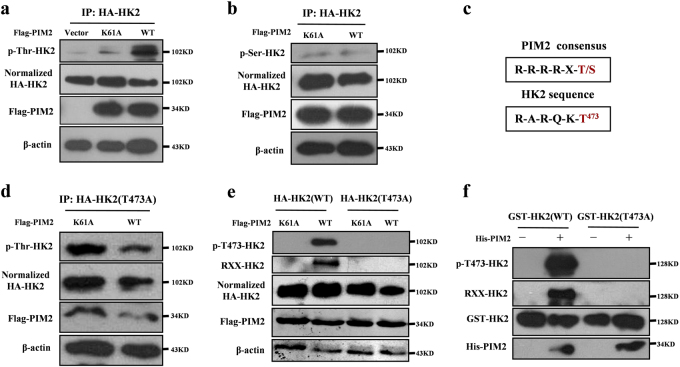
PIM2 phosphorylates HK2 at Thr473. Immunoprecipitation and immunoblotting analyses were performed with the indicated antibodies. **a**, **b** HEK293T cells were overexpressed the indicated HA-tagged HK2 and Flag-tagged PIM2 (WT or K61A) proteins. Immunoprecipitation with an anti-HA antibody was performed. **c** A putative PIM2 substrate motif was identified in HK2. **d**, **e** HEK293T cells were overexpressed the indicated HA-tagged HK2 (WT or T473A) and Flag-tagged PIM2 (WT or K61A) proteins. Immunoprecipitation with an anti-HA antibody was performed. **f** Purified GST-tagged HK2 was mixed with the indicated bacterially purified His-tagged PIM2 proteins. An in vitro kinase assay was performed

The requirement for the PIM2 kinase domain in the interaction with HK2 led us to examine whether PIM2 was responsible for direct phosphorylation of HK2. We hypothesized that PIM2 phosphorylated HK2. To test this hypothesis, we overexpressed control vector or Flag-tagged PIM2 (wild type or kinase-inactive (K61A)) with HA-tagged HK2 in HEK293T cells. Compared with the control vector or kinase-inactive PIM2, wild-type PIM2 caused an increase in HK2 phosphorylation on threonine residues (Fig. 2a), but PIM2 did not affect the phosphorylation levels of HK2 on serine residues (Fig. 2b). Moreover, we performed in silico analyses for potential PIM substrate motifs in HK2. As shown in Fig. 2c, we identified a putative PIM phosphorylation site at Thr473 in HK2. To determine whether Thr473 was phosphorylated by PIM2, we mutated this residue to alanine (T473A) and performed Co-IP assays. As shown in Fig. 2d, mutated T473A had no effect on threonine phosphorylation of HK2, suggesting that T473 contributes to the phosphorylation signals. Furthermore, phosphorylation of T473 in HK2 was confirmed by PAS antibody (phosphorylated AKT consensus sequence (RXXpT/S) antibody) and pT473-HK2 antibody. As shown in Fig. 2e, the overexpression of PIM2 increased phosphorylation of HK2 on T473, but had no effect on the T473A mutant. In vitro kinase assays showed that recombinant active PIM2 catalyzed the phosphorylation of wild-type HK2 but did not change the phosphorylation levels of the T473A mutants, which provided direct evidence that HK2 T473 was phosphorylated by PIM2 (Fig. 2f), AKT1 [9] as a positive control (Supplementary Fig. 3a). AKT1 also interacted with HK2 in MCF-7 cells (Supplementary Fig. 2d). Moreover, phosphorylation of HK2 at T473 did not affected interaction with PIM2 (Supplementary Fig. 2b). PHLPP was reported to interact with HK2 and AKT [17]. To determine whether PIM2 formed a complex with PHLPP, we performed a Co-IP assay. As shown in Supplementary Fig. 2C, PIM2 did not bind to PHLPP. Moreover, we also used AKT and PIM2 inhibitors to test effects on HK2 Thr473 phosphorylation. As shown in Supplementary Fig. 3b, AKT and PIM2 inhibitors decreased phosphorylation level of HK2 on Thr473, respectively. PIM2 inhibitor also inhibited HK2 Thr473 phosphorylation in SW480 colon cancer cells (Supplementary Fig. 3c). To determine whether AKT pathway was involved in PIM2-mediated HK2 Thr473 phosphorylation, we used inhibitor or siRNA to abrogate AKT pathway. As expected, AKT inhibitor or siRNA unchanged HK2 Thr473 phosphorylation level in PIM2-overexpressing MCF-7 cells, indicating that PIM2-mediated HK2 Thr473 phosphorylation was independent of AKT pathway (Supplementary Fig. 3d, e). Thus, our results suggested that the T473 residue of HK2 was phosphorylated by PIM2.

### PIM2 regulates HK2 protein stability via a CMA pathway

**Fig. 3:**
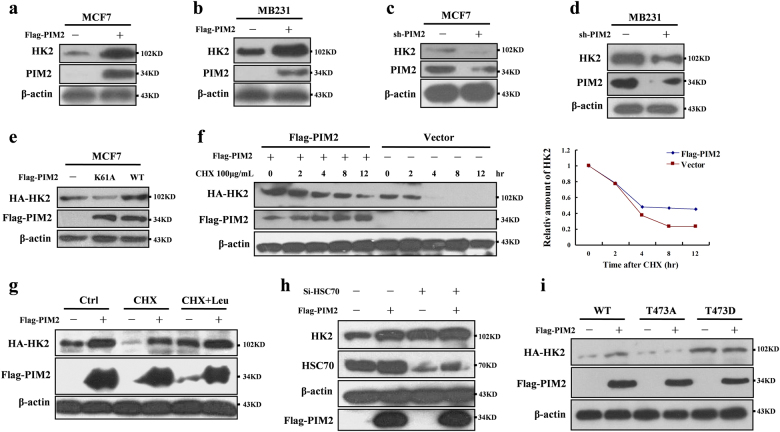
PIM2 regulates HK2 protein stability via a CMA pathway. Immunoblotting analyses were performed with the indicated antibodies. **a**, **b** MCF-7 or MB-231 cells were overexpressed the indicated Flag-tagged PIM2 proteins. Total cell lysates were prepared. **c**, **d** MCF-7 or MB-231 cells were knocked down PIM2 with shRNA. Total cell lysates were prepared. **e** MCF-7 cells were overexpressed the indicated Flag-tagged PIM2 (WT or K61A) proteins. Total cell lysates were prepared. **f** MCF-7 cells with overexpression of the indicated both Flag-tagged PIM2 and HA-tagged HK2 proteins were treated with CHX for indicated time. Total cell lysates were prepared. **g** MCF-7 cells with overexpression of the indicated both Flag-tagged PIM2 and HA-tagged HK2 proteins were treated with CHX or CHX + Leu for 12 h. Total cell lysates were prepared. **h** MCF-7 cells with HSC70 knocked down were overexpressed the indicated Flag-tagged PIM2 proteins. Total cell lysates were prepared. **i** MCF-7 cells were overexpressed the indicated both Flag-tagged PIM2 and HA-tagged HK2 (WT, T473A, or T473D) proteins. Total cell lysates were prepared

To determine the functions of PIM2-mediated HK2 phosphorylation, we examined the effect of this phosphorylation on HK2 protein stability. Overexpressed PIM2 caused the accumulation of HK2 proteins in MCF-7 and MB-231 cells (Fig. 3a, b). Conversely, PIM2 knockdown resulted in a decrease of HK2 protein in MCF-7 and MB-231 cells (Fig. 3c, d). To determine whether PIM2 stabilized HK2 by T473 phosphorylation, we overexpressed PIM2 (wild type or K61A) in MCF-7 cells. As shown in Fig. 3e, PIM2 promoted HK2 protein stability, depending on its kinase activity. To further confirm this regulation, we transfected MCF-7 cells with PIM2, and treated the cells with a protein synthesis inhibitor-cycloheximide (CHX), to examine the degradation rate of HK2. As shown in Fig. 3f, the HK2 degradation was significantly slower in overexpressing PIM2 cells than in control cells. A study reported that HK2 degradation was regulated by a CMA pathway [18]. To determine whether PIM2 regulated HK2 protein stability through a CMA pathway, transfected MCF-7 cells were treated with leupeptin, an inhibitor of lysosomal proteases. As shown in Fig. 3g, PIM2-mediated HK2 protein stability increases were completely blocked in the leupeptin-treated cells. Because HSC70 was a critical component in the CMA pathway, we knocked down HSC70 and measured HK2 protein levels in MCF-7 cells. Knockdown of HSC70 caused significant accumulation of HK2 (Supplementary Fig. 4a), and PIM2-mediated HK2 protein stability was inhibited (Fig. 3h). But PIM2 unchanged the ubiquitination level of HK2 (Supplementary Fig. 4b). To determine whether T473 phosphorylation affected HK2 protein stability, we generated mutant HA-tagged HK2 (T473A or T473D). As shown in Fig. 3i, HA-tagged HK2 (T473A) was expressed at a level significantly lower than HA-tagged HK2 (WT or T473D) in MCF-7 cells, indicating that T473 phosphorylation was responsible for enhancing the HK2 protein stability. We concluded that PIM2 regulated HK2 protein stability through a CMA-mediated protein degradation pathway.

### PIM2 expression is positively correlated with pT473-HK2 in human breast cancer

**Fig. 4:**
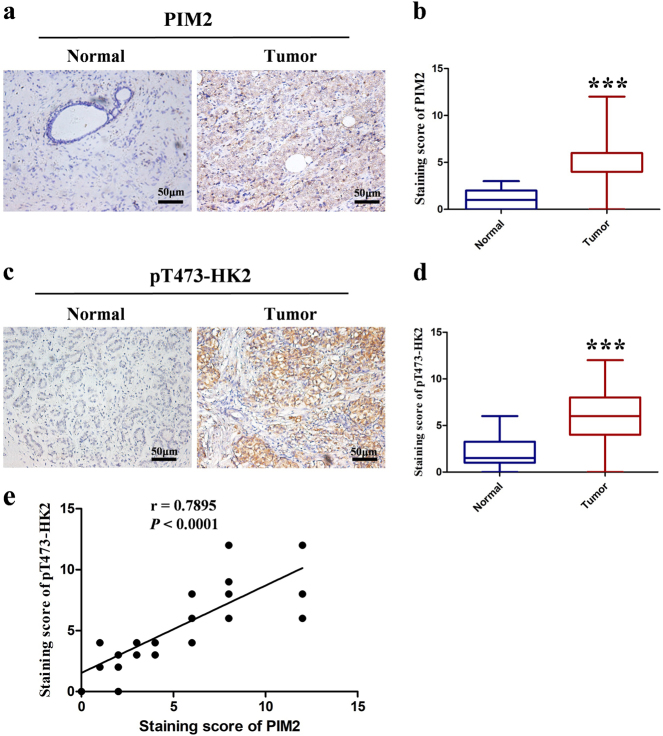
PIM2 expression is positively correlated with pThr473-HK2 in human breast cancer. **a**, **c** Representative histopathologic sections of human breast normal and cancer tissues were stained with PIM2 or pThr473-HK2 antibodies. Scale bars, 50 μm. **b**, **d** Semi-quantitative immunohistochemical analysis of human breast normal and cancer tissues for PIM2 or pThr473-HK2. The experiments were tested with paired *t*-test, ***p < 0.001. **e** Pearson correlative analysis of semi-quantitative staining scores for PIM2 and pThr473-HK2. The standard curve was drawn by linear regression of the correlation scores. Correlation is shown using *r* and significance was determined using a Spearman correlation, ***p < 0.001

To determine the expressions levels of PIM2 and pT473-HK2 in breast cancer tissues, we performed immunohistochemistry assays in 10 cases of normal breast tissues and 46 cases of breast cancer tissues. Obviously, immunohistochemical staining revealed that PIM2 (Fig. 4a, b) and pT473-HK2 (Fig. 4c, d) were strongly expressed in breast cancer tissues but had reduced expression in normal breast tissues. Consistent with these data, we found that the PIM2 and pT473-HK2 protein levels were significantly correlated with the tumor size and stage of breast tumors, but not with other factors (Table 1). Moreover, we detected the correlation between PIM2 and pT473-HK2 expression (Fig. 4e). Thus, these results indicated that the protein levels of PIM2 and pT473-HK2 were positively correlated in human breast cancer tissues, and could predict more malignant tumor characteristics.

**Table 1:** Analysis of correlation between PIM2 or pThr473-HK2 protein levels and clinicopathological parameters of breast cancer patients

		PIM2 expression				pT473-HK2 expression		
Variable	*n*	Low	High	*p-*Value	*n*	Low	High	*p*-Value
Age				0.735				0.643
≤50 years	18	6	12		18	7	11	
>50 years	28	8	20		28	9	19	
Tumor size				0.002				0.013
≤2 cm	20	11	9		20	11	9	
>2 cm	26	3	23		26	5	21	
TNM stage				0.001				0.008
I–II	22	12	10		22	12	10	
III–IV	24	2	22		24	4	20	
ER status				0.956				0.227
+	26	8	18		26	11	15	
−	20	6	14		20	5	15	
PR status				0.073				0.705
+	27	11	16		27	10	17	
−	19	3	16		19	6	13	
HER2				0.803				0.852
+	21	6	15		21	7	14	
−	25	8	17		25	9	16	

### Phosphorylation of HK2 by PIM2 promotes glycolysis and glucose starvation-induced autophagy

**Fig. 5:**
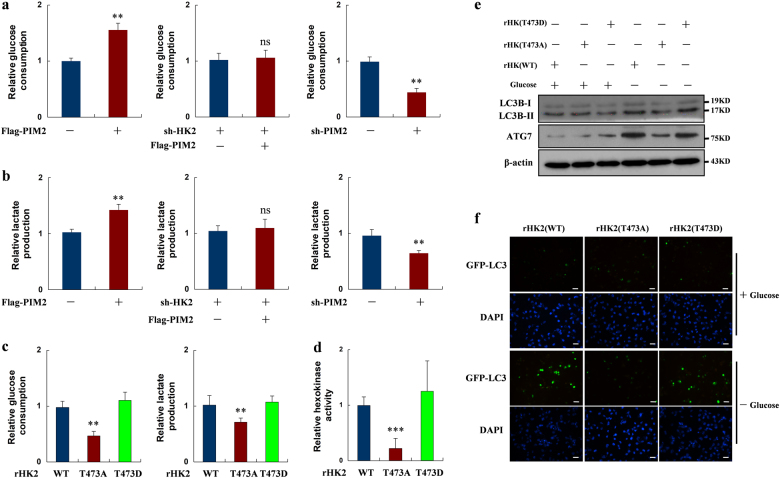
Phosphorylation of HK2 by PIM2 promotes glycolysis and glucose starvation-induced autophagy. **a**, **b** MCF-7 cells were transfected with an empty vector or Flag-tagged PIM2, shHK2, or control shRNA followed by Flag-tagged PIM2, or sh-PIM2 or control shRNA. Two days after transfection, the medium was replaced by serum-free medium for another 16 h culture. Medium were collected for analysis of glucose consumption or lactate production, which was normalized by corresponding protein amounts. **c** Stable expressing rHK2 (WT, T473A, or T473D) MCF-7 cells were re-plated in a six-well plate. After 1 day, the medium was replaced by serum-free medium for another 16 h culture. Medium were collected for analysis of glucose consumption (left) or lactate production (right), which was normalized by corresponding protein amounts. **d** HA-tagged HK2 (WT, T473A, or T473D) proteins were accumulated by IP assay in stable expressing rHK2 (WT, T473A or T473D) MCF-7 cells. Hexokinase kinase activity was measured, which was normalized by corresponding protein amounts. **e** Stable expressing rHK2 (WT, T473A, or T473D) MCF-7 cells were cultured in the presence or absence of glucose-deprived medium for 6 h. Total cell lysates were prepared. **f** Stable expressing rHK2 (WT, T473A, or T473D) MCF-7 cells were overexpressed GFP-LC3 in the presence or absence of glucose-deprived medium for 6 h. Immunofluorescence was performed to analyze GFP-LC3 expression (all data represent mean ± SEM *n* = 3), *p < 0.05, **p < 0.01, ***p < 0.001

**Fig. 6:**
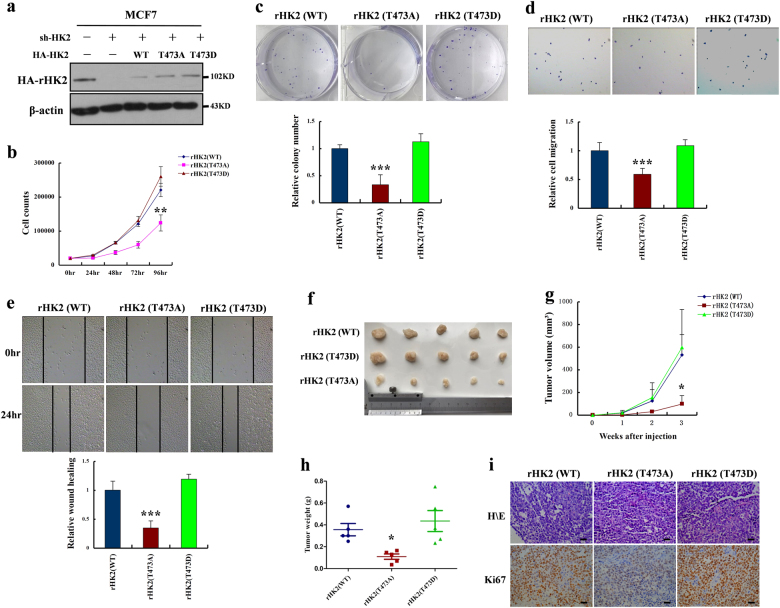
HK2 T473 phosphorylation promotes cell proliferation, cell migration, and tumor growth in breast cancer cells. **a** MCF-7 cells with HK2 depletion reconstituted stable expression of HA-tagged HK2 (WT, T473A, or T473D). Total cell lysates were prepared, and immunoblotting analyses were performed with the indicated antibodies. **b** MCF-7 cells with stable expression of rHK2 (WT, T473A, or T473D) were seeded in a 24-well plate. Cell numbers were counted every 24 h for 4 days. **c–e** MCF-7 cells with stable expression of rHK2 (WT, T473A, or T473D) were seeded in a six-well plate. Clone formation, wound healing assay, and cell invasion assays were performed. **f–h** MCF-7 cells with stable expression of rHK2 (WT, T473A, or T473D) were subcutaneously injected into nude mice. After 3 weeks, the mice were sacrificed and dissected at the endpoint. Tumor growth and weight were examined. **i** Representative images of H/E staining and Ki67 staining of tumor samples (scale bar, 20 μm) (all data represent mean ± SEM *n* ≥ 3), *p < 0.05, **p < 0.01, ***p < 0.001

HK2 is a key mediator in regulating glycolysis [5], so we hypothesized that PIM2 regulation of glycolysis depended on HK2. To validate our hypothesis, we overexpressed PIM2 in MCF-7 cells. Interestingly, overexpression of PIM2 increased both glucose consumption and lactate production in MCF-7 cells (Fig. 5a, b). In contrast, knockdown of PIM2 decreased glycolysis in MCF-7 cells (Fig. 5a, b). To determine whether HK2 was required for PIM2 regulation of glycolysis, we knocked down HK2 in MCF-7 cells and then overexpressed PIM2 in the same cells. PIM2-increased glucose consumption and lactate production were blocked when HK2 was knocked down (Fig. 5a, b). To determine the effects of PIM2-mediated HK2 phosphorylation at the T473 residue on glycolysis, we established stable HK2 shRNA MCF-7 cells with reconstituted expression of HA-tagged HK2 (WT, T473A, or T473D) (Fig. 6a). As shown in Fig. 5c, the T473A HK2 mutant decreased both glucose consumption and lactate production. Moreover, the T473A HK2 mutant had lower hexokinase activity in MCF-7 cells (Fig. 5d). Thus, our data suggested that phosphorylation of HK2 on T473 was involved in regulating its enzymatic activity and glycolysis.

HK2 has been reported to positively regulate glucose starvation-induced autophagy [7]. To determine whether phosphorylation of HK2 by PIM2 had an effect on autophagy, we made glucose starvation to stimulate stable overexpressing HA-rHK2 (WT, T473A, or T473D) MCF-7 cells. As expected, the T473A mutant HK2 decreased autophagy compared with wild-type HK2 or T473D HK2, but there was no effect in the presence of glucose (Fig. 5e). To further determine the effect of this phosphorylation on autophagic flux, we overexpressed GFP-LC3 in stable MCF-7 cell lines. As shown in Fig. 5f, the T473A mutant HK2 had a lower GFP-LC3 expression than HK2 (WT or T473D) during glucose starvation. Taken together, our data demonstrated that phosphorylation of HK2 by PIM2 was required for autophagy during glucose starvation.

### HK2 T473 phosphorylation promotes cell proliferation, cell migration, and tumor growth in breast cancer cells

To investigate the role of HK2 T473 phosphorylation in breast cancer cell proliferation, cell migration, and tumor growth, we used stable HK2 shRNA MCF-7 cells with reconstituted expression of HA-tagged HK2 (WT, T473A, or T473D) (Fig. 6a). We found that stably expressing rHK2 (T473A) MCF-7 cells proliferated lowly compared with rHK2 (WT or T473D) MCF-7 cells (Fig. 6b, c), indicating a growth advantage conferred by the phosphorylation at Thr473. In addition, phosphorylation of HK2 T473 by PIM2 enhanced cell migration which was determined by cell-scratch tests and transwell migration assays (Fig. 6d, e). Moreover, phosphorylation of HK2 on Thr473 also promoted cell proliferation in MCF-10A cell lines (Supplementary Fig. 5a). To determine whether HK2 T473 phosphorylation also rendered a growth advantage to tumor cells in vivo, we performed xenograft studies. Stably rHK2 (WT, T473A, or T473D) MCF-7 cells were subcutaneously injected into the nude mice. As shown in Fig. 6f, tumors from rHK2 (T473A) MCF-7 cells grew significantly slower than those from rHK2 (WT or T473D) MCF-7 cells. As a result, rHK2 (T473A) MCF-7 cells gave rise to significantly smaller tumors than those rHK2 (WT or T473D) cells as measured by tumor volume (Fig. 6g) and weight (Fig. 6h). By Ki67 staining, we further confirmed that rHK2 (T473A) cells were less proliferative than rHK2 (WT or T473D) cells in vivo (Fig. 6i). Together, the results showed that phosphorylation of HK2 by PIM2 was important for breast cancer progression in vivo.

### Phosphorylation of HK2 by PIM2 promotes breast cancer cell paclitaxel resistance

**Fig. 7:**
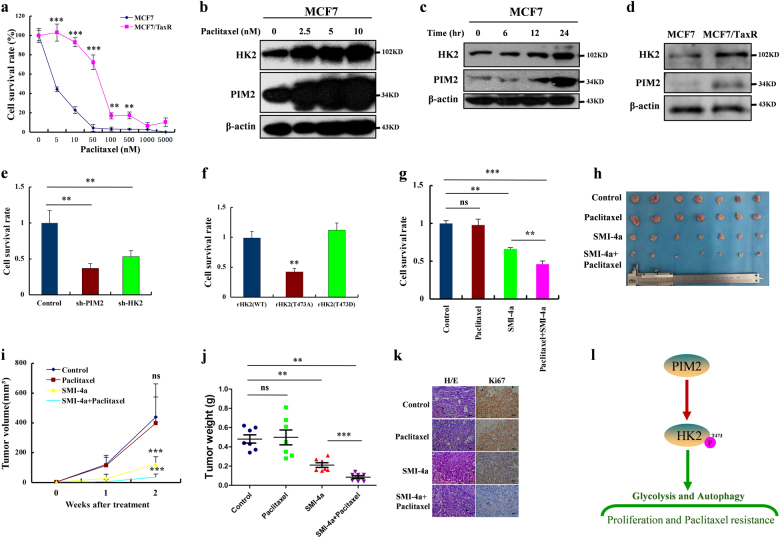
Phosphorylation of HK2 by PIM2 promotes breast cancer cell paclitaxel resistance. **a** MCF-7 and MCF-7/TaxR cells were treated for 2 days with increasing concentrations of paclitaxel, and their proliferation rates were measured by cell counting. **b** MCF-7 cells were treated with 0, 2.5, 5, 10 nM paclitaxel for 2 days. Total cell lysates were prepared, and immunoblotting analyses were performed with the indicated antibodies. **c** MCF-7 cells were treated with 10 nM paclitaxel for 0, 6, 12, and 24 h. Total cell lysates were prepared, and immunoblotting analyses were performed with the indicated antibodies. **d** Total cell lysates from MCF-7 and MCF-7/TaxR cells were prepared, and immunoblotting analyses were performed with the indicated antibodies. **e** MCF-7/TaxR cells with PIM2 or HK2 knocked down were treated with 10 nM paclitaxel for 2 days. Cell proliferation rates were measured by cell counting. **f** MCF-7 cells with stable expression of rHK2 (WT, T473A, or T473D) were treated with 10 nM paclitaxel for 2 days. Cell proliferation rates were measured by cell counting. **g** MCF-7/TaxR cells were treated with PBS (control), 10 nM paclitaxel, 5 nM SMI-4a, or 10 nM paclitaxel combined with 5 nM SMI-4a for 2 days. Cell proliferation rates were measured by cell counting. **h**–**j** MCF-7/TaxR cells were injected into nude mice. After 1 week, the mice were randomly assigned to four groups: PBS (control), paclitaxel, SMI-4a, and SMI-4a combined with paclitaxel, and given intraperitoneally three times each week for treatment. After 2 weeks of treatment, the mice were sacrificed, and the tumor weight was measured. Tumor volume was measured during the tumor growth for 3 weeks. **k** The tumor tissues were examined by hematoxylin and eosin (H/E) and Ki67 (scale bar, 20 µm) (all data represent mean ± SEM *n* ≥ 3), *p < 0.05, **p < 0.01, ***p < 0.001

Breast cancer is one of the most malignant tumors among women, and a leading cause of mortality worldwide [19]. The currently available targeted therapy is still chemotherapy, to which many patients are highly resistant [19]. Paclitaxel belongs to the class of chemotherapeutic drugs that are commonly used as single agents or in combination with anthracyclines or radiotherapy for the treatment of breast cancers [19]. For this purpose, we generated paclitaxel-resistant breast cancer cell lines (MCF-7/TaxR) by continuous exposure of the parental drug-sensitive MCF-7 cells to increasing concentrations of the drug until resistance to paclitaxel occurred (Fig. 7a). To determine a possible role of PIM2 in paclitaxel resistance, we studied the expression levels of PIM2 and its target HK2 in the breast MCF-7 or MCF-7/TaxR cells in response to paclitaxel treatment. As shown in Fig. 7b, PIM2 and HK2 expressions were upregulated in a paclitaxel concentration-dependent manner in MCF-7 cells, while the expression levels of PIM2 and HK2 had no changes in the MCF-7/TaxR cells (Supplementary Fig. 5b). Moreover, PIM2 and HK2 expression were also upregulated in response to 10 nM paclitaxel in a time-dependent manner (Fig. 7c). As we expected, PIM2 and HK2 were expressed more in MCF-7/TaxR cells than in MCF-7 cells (Fig. 7d). To investigate if PIM2 and HK2 conferred paclitaxel resistance in breast cancer cells, we analyzed the effects of PIM2 and HK2 knockdown on the cell proliferation rates of MCF-7/TaxR cells following paclitaxel treatment. Knockdown of PIM2 and HK2, respectively, resulted in a decrease in cell proliferation rate of MCF-7/TaxR cells (Fig. 7e). Furthermore, stably rHK2 (T473A) MCF-7 cells also had decreased resistance to paclitaxel compared with rHK2 (WT or T473D) MCF-7 cells, which suggested phosphorylation of HK2 at T473 played a crucial role in the resistance of breast cancer cells (Fig. 7f). Because paclitaxel-resistant cells had increased PIM2 expression, we explored whether a PIM2-specific inhibitor, SMI-4a, could sensitize paclitaxel-resistant cells to paclitaxel. We found that the combination of paclitaxel and SMI-4a synergistically inhibited the growth of MCF-7/TaxR cells (Fig. 7g). Consistently, 2-DG as a HK2 inhibitor had same effects with SMI-4a on inhibiting the growth of MCF-7/TaxR cells (Supplementary Fig. 5c). To further confirm these results in vivo, we used combination treatments of paclitaxel and SMI-4a in a xenograft nude mouse model. As expected, compared with the control group, the combination treatment was more efficient in the inhibition of MCF-7/TaxR tumor volume (Fig. 7h, i). Similarly, combined treatment decreased tumor weight compared with either of the single agents (Fig. 7j). Ki67 staining showed that the combination treatments of paclitaxel and SMI-4a had a significant inhibitory effect on proliferation of MCF-7/TaxR cells (Fig. 7k). No apparent body weight loss or toxicity was observed in any treatment group (Supplementary Fig. 5d). These results further suggested that PIM2-mediated HK2 phosphorylation contributed to paclitaxel resistance.

## Discussion

Aerobic glycolysis, also named the Warburg effect, provides ATP and intermediary metabolic products to support tumorigenesis [1]. High aerobic glycolysis is applied clinically for diagnosis and monitoring of cancers by imaging uptake of 2-^18^F-deoxyglucose with positron emission tomography (PET) imaging [20]. HK2 is the first step of glycolysis, which converts glucose to G6P using cytosolic ATP [2]. Although HK2 is upregulated in breast cancer [4], the molecular mechanisms of HK2 involved in post-translational modifications are not clear. Here, we discovered a novel interaction between PIM2 and HK2 proteins, both of which were upregulated and correlated with each other in human breast cancer tissues. Our biochemical analyses demonstrated that PIM2 bound to HK2 and phosphorylated HK2 at T473. The HK2 T473 phosphorylation might be involved in regulating aerobic glycolysis of breast cancer cells. Indeed, evidences showed that phosphorylation of HK2 by PIM2 promoted glycolysis in MCF-7 cells. Moreover, PIM2 regulation of glycolysis was HK2-dependent. Thus, PIM2 may be a positive regulator of HK2 by post-translational modification.

PIM2 is a Thr/Ser kinase with numerous substrates, such as BAD, p21, p27, MDM2, and TSC2, which are regulated by post-translational modifications [11]. Moreover, PIM2 plays an important role in regulating glycolysis, and promotes cancer progression [15, 21**]**. In our previous study, we found that PIM2 promoted glycolysis through phosphorylation of PKM2 at T454 [15]. In addition, a previous study showed that HK2 T473 could be phosphorylated by AKT, and this promoted mitochondrial translocation of HK2 in cardiomyocytes [9]. As a result, the outer mitochondrial membrane localization of HK2 allows access to ATP to catalyze its enzymatic reactions more efficiently. This is crucial for controlling glycolytic rates to fulfill the bioenergetics and biosynthetic needs of proliferating cells. These results are consistent with our hypothesis that PIM2-mediated HK2 phosphorylation promoted glycolysis. Our findings suggested a novel molecular mechanism involving PIM2 regulation of glycolysis by phosphorylation of HK2 at T473. The protumorigenic functions for autophagy are largely attributed to the ability to promote cancer cell survival in response to diverse stresses including glucose starvation [22]. Interestingly, we found that glucose starvation was required for HK2 T473 phosphorylation-induced autophagy.

Paclitaxel as a chemotherapeutic agent is widely used for the treatment of several types of cancers, including breast cancer [19]. Paclitaxel resistance remains the main cause of death in breast cancer therapy [19]. Although studies have been reported regarding paclitaxel resistance in breast cancer cells [19], the molecular mechanisms that mediated paclitaxel resistance remain unknown. Previous studies showed that paclitaxel-resistant cells had high glycolysis rates, which resulted in more glucose uptake and lactate production [23]. This conclusion was consistent with our experimental results that phosphorylation of HK2 by PIM2 enhanced glycolysis and contributed to the resistance of breast cancer cells to paclitaxel.

In summary, our results demonstrated that PIM2 functions as a unique and key regulator of cancer development by virtue of its coordination of glycolysis, autophagy, and paclitaxel resistance through the phosphorylation of HK2 (Fig. 7l). Our data further suggested that the PIM2-mediated HK2 phosphorylation may provide a potential therapeutic target for the treatment of breast cancer.

## Materials and methods

### Materials and cell culture

Rabbit anti-PIM2 and anti-HK2 antibodies were purchased from GeneTex. Mouse anti-phosphoserine antibody, Rabbit anti-HSC70 antibody, Rabbit anti-LC3 antibody, Rabbit anti-ATG7 antibody, and Rabbit anti-PHLPP antibody was from Abcam. Rabbit anti-phosphothreonine and PAS (phosphorylated Akt consensus sequence antibody) antibodies were obtained from Cell Signaling. Leupeptin, cycloheximide, G418, 2-DG, rabbit or mouse anti-HA, -GFP, -Flag, or β-actin antibody were from Sigma. Rabbit anti-pThr473-HK2 antibodies were generated by a small peptide (RARQKTpLEHC). Puromycin was from GBICO. Rabbit IgG and mouse IgG were from Santa Cruz Biotechnology. Rabbit anti-AKT1 antibody, goat anti-mouse and goat anti-rabbit second antibodies were purchased from Proteintech. Protein A agarose, Ni-NTA His-binding resin, and GST-binding resin were from GE Healthcare. Paclitaxel and SMI-4a were obtained from Calbiochem. MK2206 was obtained from MedChem express (MCE).

HEK293T, MCF-7, MDA-MB-231, and SW480 cell lines were purchased from ATCC and maintained in Dulbecco's modified Eagle's medium (DMEM) (Hyclone) supplemented with 10% fetal bovine serum (FBS) (GIBCO, CA, USA), 100 U/ml penicillin and 100 μg/ml streptomycin at 37 °C and 5% CO_2_. MCF-10A cell line was from ATCC and maintained in DMEM/F12 (Hyclone) supplemented with 10% FBS. Glucose starvation was carried out by culturing cells in the medium without glucose (GIBCO). Paclitaxel-resistant MCF-7 cells were developed from parental MCF-7 cells. Paclitaxel-resistant breast cancer cells named MCF-7/TaxR cells were maintained in DMEM medium supplemented with 10% FBS, 100 U/ml penicillin and 100 μg/ml streptomycin, and 5 nM of paclitaxel.

### siRNA and transfection

Cells were plated at a six-well plate, and then the transfection procedure was performed as previously described [15]. After 24 h, cells were re-plated in indicated size plates to perform other experiments. HSC70 siRNA was 5′- CUGUCCUCAUCAAGCGUAA-3′ [24]. AKT1 siRNA was 5′- GCUACUUCCUCCUCAAGAA-3′ [25].

### DNA constructs and mutagenesis

PCR-amplified human PIM1, PIM2, PIM3, HK2, LC3, and AKT1 were cloned into pcDNA3.0/HA, pFlag-CMV4, pEGFP-C1, pet28a, or pGEX-4T-1. pcDNA3.0/HA-HK2 (T473A or T473D) and pFlag-CMV4-PIM2 (K61A) were generated by using QuickChange site-directed mutagenesis kit (Stratagene, #200519). The HK2 shRNA was generated with oligonucleotide 5-CACGATGAAATTGAACCTGGT-3 [5], the PIM2 shRNA was generated with oligonucleotide 5-CTCGAAGTCGCACTGCTAT-3 [15], and the lentiviruses were produced by GenePharma Company (China).

### Purification of recombinant proteins

The WT or mutant GST-HK2, His-PIM2, and His-AKT1 were expressed in *E. coli* BL21 (DE3), and purified as described previously [15].

### Immunoprecipitation and GST-pulldown assays

Extraction of protein with immunoprecipitation (IP) buffer (Beyotime, China) from cultured cells was followed by IP and immunoblotting with indicated antibodies as described previously [15]. Protein A agarose, Ni-NTA His-binding resin, and GST-binding resin were incubated with cell lysates or purified proteins for more than 8 h, and washed with the lysis buffer for five times.

### In vitro kinase assays

The bacterially purified recombinant GST-HK2 (WT or T473A) (2 μg) were incubated with His-PIM2 (0.2 μg) in 50 μl kinase buffer at 37 °C for 30 min [15]; His-AKT1 was used as a positive control in AKT kinase buffer at 30 °C for 20 min [26]. The reactions were stopped by the addition of sodium dodecyl sulfate polyacrylamide gel electrophoresis (SDS-PAGE) loading buffer and heated to 100 °C. The reaction mixtures were then used for SDS-PAGE.

### Confocal immunofluorescence microscopy

Confocal immunofluorescence microscopy was performed as described previously [15, 27**]**.

### Glucose consumption and lactate production

Cells were reseeded in six-well plates. After 6 h, the medium was changed with nonserum DMEM. The culture medium was collected after incubated for 16 h to measurement of glucose and lactate levels [15]. Glucose levels were determined using a glucose (GO) assay kit (Sigma, #GAGO20-1KT), and lactate levels were determined using a lactate assay kit (Biovision, # K627-100). These readouts were normalized to corresponding protein amounts.

### Tissue collection and immunohistochemical staining

This study was approved by the Ethics Committee of Weifang Medical University. We obtained the 10 cases of normal breast tissues and the 46 breast cancer tissues from affiliated hospital of Weifang Medical University (Weifang, China), and were agreed by all patients. The sections were stained with indicated antibodies, and staining of PIM2 and HK2 was scored by two independent researchers based on the percentage of positive cells and staining intensity. The percentage of positive staining was graded as 0 = 0–5%, 1 = 6–25%, 2 = 26–50%, 3 = 51–75%, and 4 = 76–100%, and a staining intensity score (0 = none, 1 = weak, 2 = moderate, and 3 = strong). The staining grade was stratified as absent (score 0), weak (score 1 to 4), moderate (score 5 to 8), or strong (score 9 to 12). Tumors in a score of >4 were classified as having high expression, and tumors in a score of ≤4 were classified as having low expression.

### Knocking down and putting back stable cell lines

The shHK2 lentiviruses were added, with polybrene, to the appropriate MCF-7 cells in a six-well plate. Twelve hours later, the medium was changed to fresh medium. Finally, 24 h after infection the cells were selected in the presence of puromycin (1 μg/ml) for 2 weeks. The cells were re-plated into the wells of a 96-well plate containing 150 μl DMEM supplemented with 30% serum and 0.5 μg/ml puromycin. After 2 weeks of growth, individual clones were expanded. The shHK2 MCF-7 cells were transfected with pcDNA3/HA-HK2 (WT, T473A, or T473D). After 48 h incubation, we used G418 (1 mg/ml) to screen HK2 stable expression MCF-7 cells for 2 weeks. The single stable cells were selected by reseeded into 96-well plates with 30% serum and 0.5 mg/ml G418.

### Measurement of hexokinase kinase activity

We performed IP assays to enrich HA-rHK2 (WT, T473A, or T473D) proteins from stable HK2-overexpressing MCF-7 cell lines. The accumulated proteins were washed five times by phosphate-buffered saline (PBS), and hexokinase kinase activity was measured using Hexokinase Colorimetric Assay Kit (Sigma, # MAK091-1KT) according to the manufacturer’s instructions. The results were normalized to corresponding protein amounts.

### Cell proliferation analysis

The cells were re-plated in triplicates onto 24-well plates and counted in the indicated days, and cell numbers were counted every 24 h [15].

### Clone formation, wound healing assay, and cell invasion assay

The cells were seeded onto six-well plates at a concentration of 200 cells in 2 ml culture medium per well and cultured at 37 °C and 5% CO_2_ for 12–14 days. Cells were fixed by 4% paraformaldehyde, and then treated with crystal violet staining solution, and cells were photographed [28]. For wound healing assay, cells were cultured in six-well plates till a monolayer was formed. The cells were then wounded by scratching with yellow pipette tips, and scratches were photographed after 24 h. Cell invasion assay were performed as previously described [29].

### Xenograft tumor studies

All experiments using animals were in accordance with the guidelines published by the Animal Ethics Committee of Weifang Medical University. Sixty-day release pellets containing 17β-estradiol (0.18 mg) (Innovative Research of America, Sarasota, FL) were implanted subcutaneously 2 days before injecting the cells. BALB/c nude mice (female 4-week-old) were injected subcutaneously with 5 × 10^6^ rHK2 (WT, T473A, or T473D) stable expression MCF-7 cells. Tumor volume was measured during the tumor growth for 3 weeks. Tumor volume was calculated according to the following formula: tumor volume = (length × width^2^)/2. After 3 weeks, the mice were killed, and tumors were weighed.

For paclitaxel-resistant experiments, BALB/c nude mice (female 4-week-old) were injected subcutaneously with 1 × 10^7^ MCF-7/TaxR cells. After 1 week, the mice were randomly assigned to four groups: PBS (control), paclitaxel (10 mg/kg) alone, SMI-4a (60 mg/kg) alone, and SMI-4a combined with paclitaxel, and given intraperitoneally three times each week for treatment. Tumor volume was measured during the tumor growth for 3 weeks. After 2 weeks treatment, the mice were sacrificed, and the body and tumor weights were measured.

### Statistical analysis

All statistical analyses were done using SPSS software version 17.0 (Chicago, IL) or Graphpad Prism 5.0 software, and presented as mean ± SEM Statistical analyses were performed by using the two-tailed unpaired Student’s *t*-test. Differences were considered to be statistically significant at *p* < 0.05. *p < 0.05, **p < 0.01, ***p < 0.001. Statistical significance was displayed as *p < 0.05, and n.s. was not significant.

## Electronic supplementary material

SUPPLEMENTARY FIGURES and LEGENDS

Supplementary Table 1
