# Correction: PIM2-mediated phosphorylation of hexokinase 2 is critical for tumor growth and paclitaxel resistance in breast cancer

**DOI:** 10.1038/s41388-019-0982-4

**Published:** 2019-09-11

**Authors:** Tingting Yang, Chune Ren, Pengyun Qiao, Xue Han, Li Wang, Shijun Lv, Yonghong Sun, Zhijun Liu, Yu Du, Zhenhai Yu

**Affiliations:** 1 Department of Reproductive Medicine, Affiliated Hospital of Weifang Medical University, Weifang, Shandong PR China; 2 Department of Pathology, Affiliated Hospital of Weifang Medical University, Weifang, Shandong PR China; 3 Department of Medical Microbiology, Weifang Medical University, Weifang, Shandong PR China

**Keywords:** Post-translational modifications, Cancer metabolism

**Correction: Oncogene**

10.1038/s41388-018-0386-x

In the original version of this article the authors noted that they accidentally used the same image in Fig. 6e: the image for 24 h rHK2 (T473D) was also used for 24 h rHK2 (WT). The amended version of Fig. 6e is provided below. This change does not affect the results or conclusions reported in the manuscript.

**Fig. 6:**
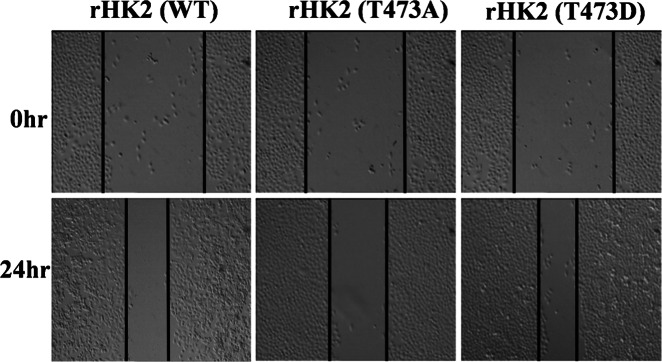
**c**–**e** MCF-7 cells with stable expression of rHK2 (WT, T473A, or T473D) were seeded in a six-well plate. Clone formation, wound healing assay, and cell invasion assays were performed

The figure has now been corrected in both the PDF and HTML versions of the Article.

