# Supplementary material for: PIM2-mediated phosphorylation of hexokinase 2 is critical for tumor growth and paclitaxel resistance in breast cancer
Source: Oncogene. 2018 Jul 9;37(45):5997–6009. doi: 10.1038/s41388-018-0386-x (PMC6224402; doi:10.1038/s41388-018-0386-x)
Supplement: Supplementary file 1 — SUPPLEMENTARY FIGURES and LEGENDS [file 41388_2018_386_MOESM1_ESM.pdf]

SUPPLEMENTARY FIGURES and LEGENDS

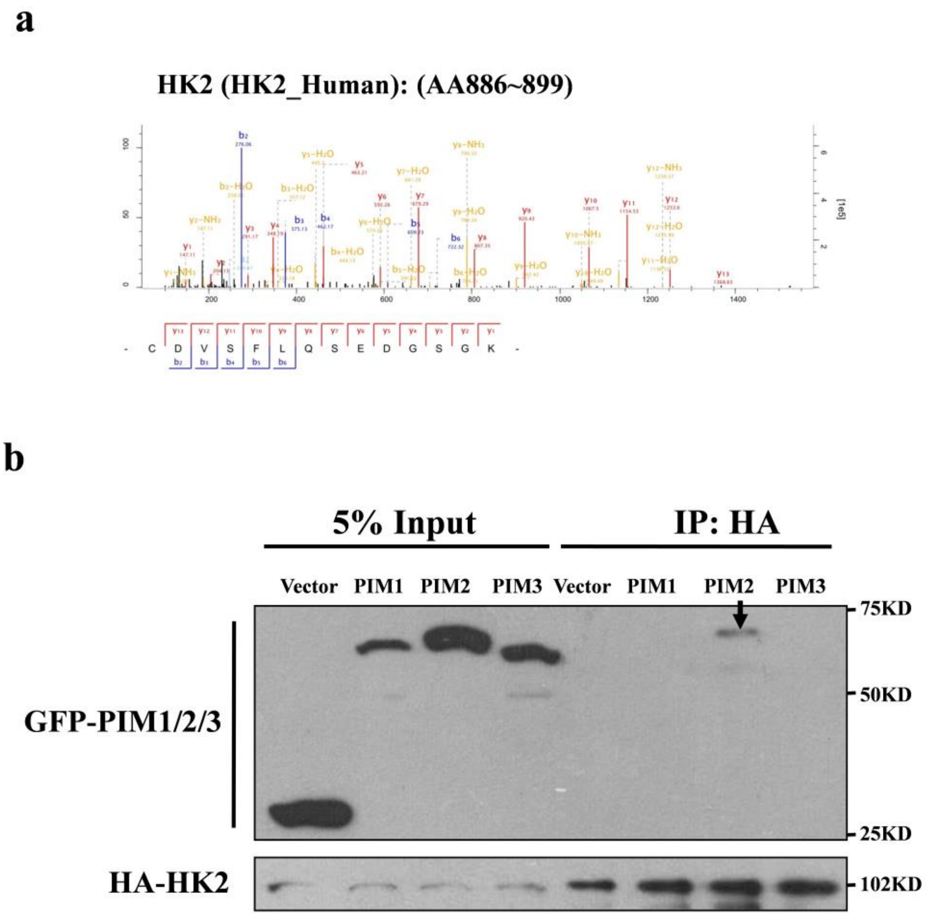

Supplementary Fig. 1

a MS-MS spectra of representative HK2 peptide.

b HEK293T cells were overexpressed HA-tagged HK2 with GFP-tagged PIM1, PIM2, or PIM3. Immunoprecipitations with an anti-HA antibody were performed, and immunoblotting analyses were performed with the indicated antibodies.

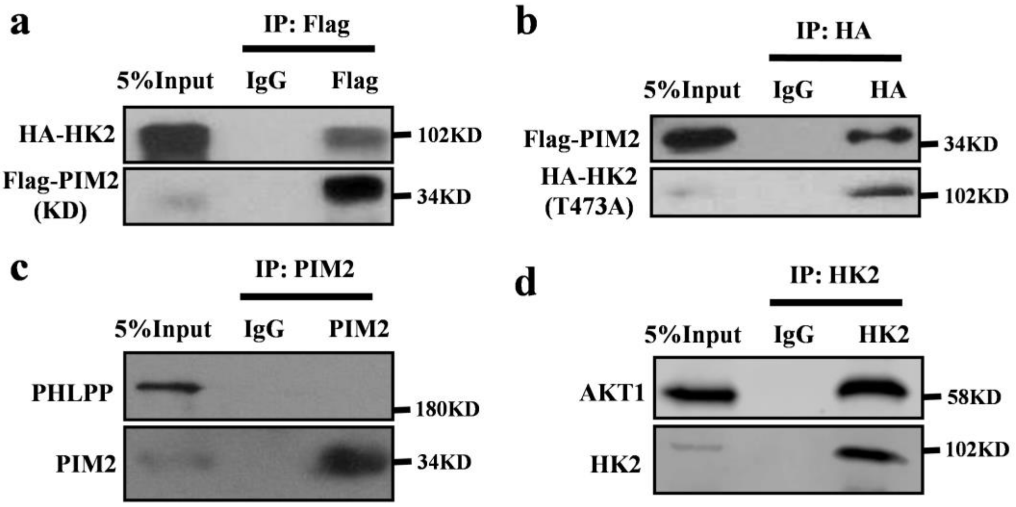

Supplementary Fig. 2

a HEK293T cells were overexpressed HA-tagged HK2 with Flag-tagged PIM2 (Kinase dead). Immunoprecipitations with an anti-Flag antibody were performed, and immunoblotting analyses were performed with the indicated antibodies.

b HEK293T cells were overexpressed HA-tagged HK2 (T473A) with Flag-tagged PIM2. Immunoprecipitations with an anti-HA antibody were performed, and immunoblotting analyses were performed with the indicated antibodies.

c Immunoprecipitations with an anti-PIM2 antibody were performed using cell lysate from MCF-7 cells, and immunoblotting analyses were performed with the indicated antibodies.

d Immunoprecipitations with an anti-HK2 antibody were performed using cell lysate from MCF-7 cells, and immunoblotting analyses were performed with the indicated antibodies.

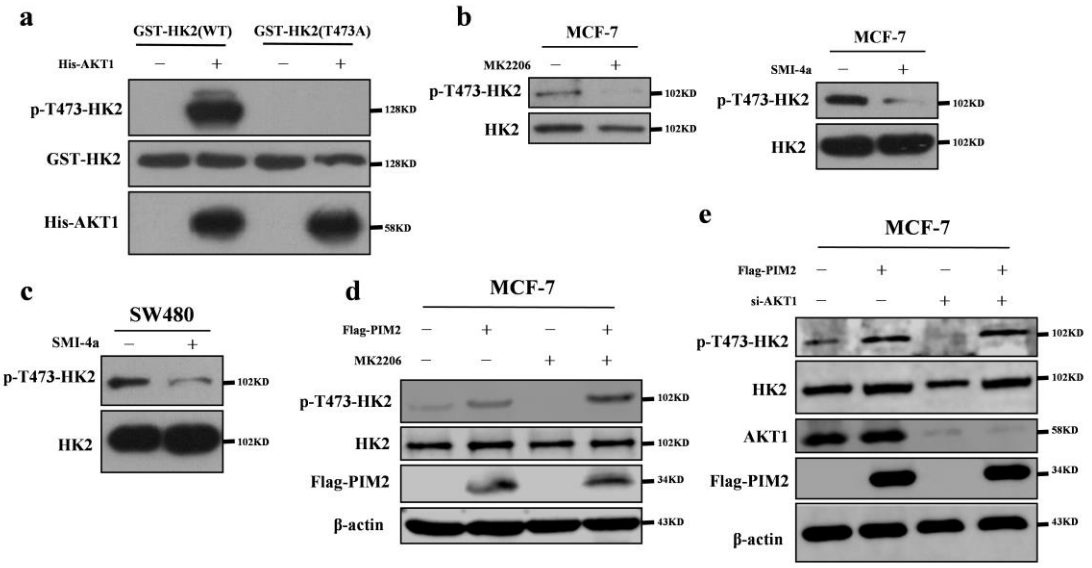

Supplementary Fig. 3

a Purified GST-tagged HK2 was mixed with the indicated bacterially purified His-tagged AKT1 proteins. An in vitro kinase assay was performed.

b MCF-7 cells were treated with MK2206 (2 μM) and SMI-4a (5nM) for one day, repectively. The immunoblotting analyses were performed with the indicated antibodies.

c SW480 cells were treated with SMI-4a (5nM) for one day. The immunoblotting analyses were performed with the indicated antibodies.

d PIM2 overexpressing MCF-7 cells were treated with MK2206 (2 μM) for one day. The immunoblotting analyses were performed with the indicated antibodies.

e AKT1 was knocked down by siRNA in PIM2 overexpressing MCF-7 cells. The immunoblotting analyses were performed with the indicated antibodies.

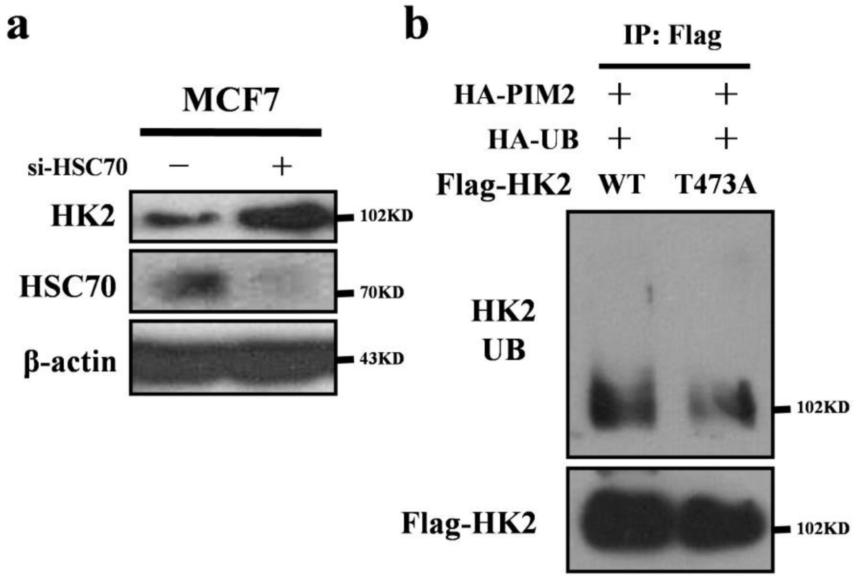

Supplementary Fig. 4

a MCF-7 cells were knocked down HSC70. Total cell lysates were prepared, and immunoblotting analyses were performed with the indicated antibodies.

b HEK293T cells were co-transfected with HA-tagged Ubiquitin and Flag-tagged HK2 (WT or T473A) in the presence or absence of HA-tagged PIM2. After 2 days transfection, cells were treated with MG132 for another 8hr incubation. Then the cell lysates were immunoprecipitated with anti-Flag antibody followed by Western blotting using the indicated antibody.

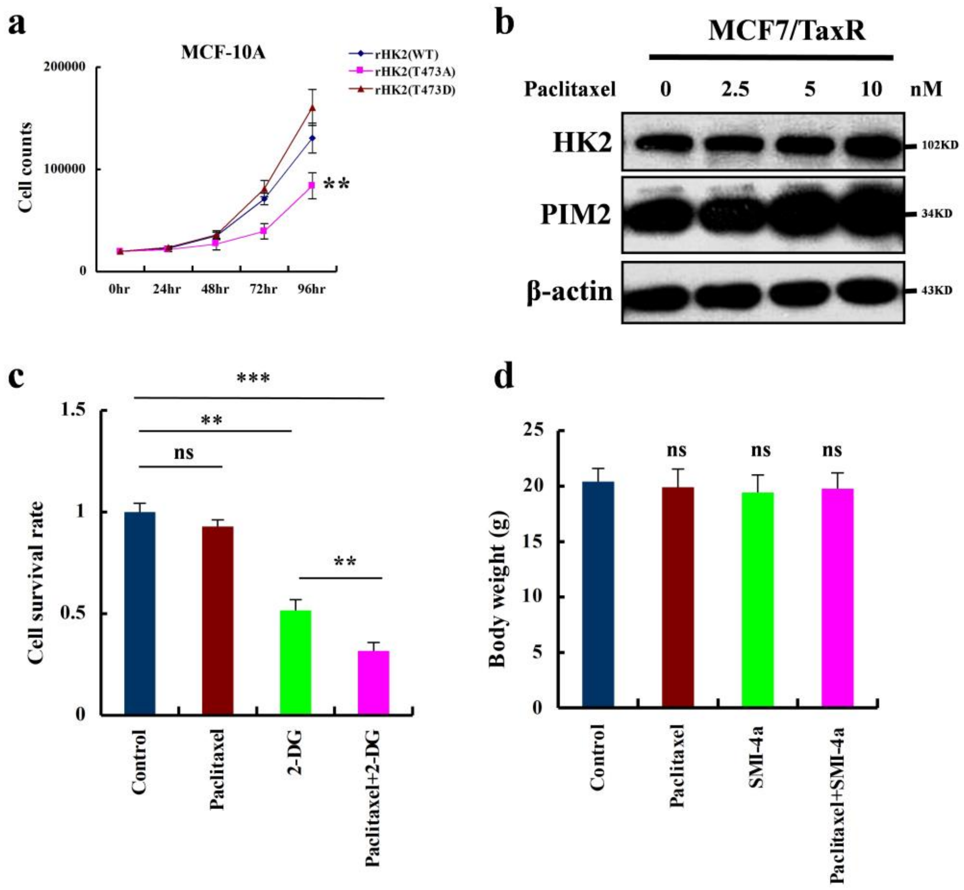

Supplementary Fig. 5

a MCF-10A cells with stable overexpression of rHK2 (WT, T473A or T473D) were seeded in a 24-well plate. Cell numbers were counted every 24hr for four days. (Data represent mean ± SEM n = 3), **p<0.01.

b MCF-7/TaxR cells were treated with 0, 2.5, 5, 10nM paclitaxel for two days. Total cell lysates were prepared, and immunoblotting analyses were performed with the indicated antibodies.

c MCF-7/TaxR cells were treated with PBS (control), 10nM paclitaxel, 0.5mM 2-DG or 10 nM paclitaxel combined with 0.5mM 2-DG for two days. Cell proliferation rates were measured by cell counting. (Data represent mean ± SEM n = 3), **p<0.01, ***p<0.001.

d The mice were sacrificed after treatment. The body weights were measured. (Data represent mean ± SEM n = 7), n.s. was not significant.
